# Isolated A1-domain *F8* large deletions are associated with favorable immune tolerance induction outcomes in children with severe hemophilia A

**DOI:** 10.1016/j.rpth.2026.106927

**Published:** 2026-08-27

**Authors:** Zhengping Li, Zhenping Chen, Jialu Zhang, Yinqi Mao, Zekun Li, Guoqing Liu, Wanru Yao, Di Ai, Wenman Wu, Runhui Wu

**Affiliations:** 1 Hemophilia Comprehensive Care Center, Hematology Department, Hematology Center, Beijing Key Laboratory of Pediatric Hematology-Oncology, Key Laboratory of Major Diseases in Children, National Key Discipline of Pediatrics (Capital Medical University), Ministry of Education, Beijing Children’s Hospital, Capital Medical University, National Center for Children’s Health, Beijing, China; 2 Cell and Gene Therapy Laboratory, Beijing Pediatric Research Institute, Beijing Children's Hospital, Capital Medical University, National Center for Children's Health, Beijing, China; 3 Department of Laboratory Medicine, Ruijin Hospital, Shanghai Jiaotong University School of Medicine, Shanghai, China; 4 State Key Laboratory of Medical Genomics, Shanghai Institute of Hematology, Ruijin Hospital, Shanghai Jiaotong University School of Medicine, Shanghai, China

**Keywords:** A1 domain, children, *F8* large deletion, immune tolerance induction, severe hemophilia A

## Abstract

**Background:**

Large *F8* deletions are generally associated with unfavorable immune tolerance induction (ITI) outcomes in children with severe hemophilia A. Whether intragenic deletion location further stratifies ITI outcomes remains unclear.

**Objectives:**

To evaluate the association between the location of *F8* large deletion and ITI outcome in children with severe hemophilia A and to exploratorily assess residual *F8* expression across patients with different deletion locations.

**Methods:**

This single-center, retrospective cohort included 34 children with severe hemophilia A, *F8* large deletions, inhibitors, and evaluable ITI outcomes. Deletions were mapped to factor VIII (FVIII) domains and categorized as isolated A1-domain or nonisolated A1-domain deletions. Exploratory *F8* mRNA and protein analyses were performed in patients with available blood samples.

**Results:**

Thirteen patients achieved ITI success or partial success, and 21 experienced ITI failure. Success or partial success was more frequent with isolated A1-domain than nonisolated A1-domain deletions (8/9 vs 5/25; *P* < .001). Higher historical peak, pre-ITI, and on-ITI peak inhibitor titers were associated with failure in univariate analyses. After multivariable adjustment, isolated A1-domain deletion remained associated with higher odds of success or partial success (adjusted odds ratio, 21.59; 95% confidence interval, 1.24-375.60; *P* = .035). Residual downstream *F8* transcripts were detected in the isolated A1-domain subgroup; their biological relevance remains uncertain.

**Conclusions:**

Intragenic location of *F8* large deletions was associated with ITI outcome in this cohort. Isolated A1-domain deletions may identify patients with a more favorable ITI prognosis. Exploratory expression findings require confirmation in larger studies using physiologically relevant models.

## Essentials

• Association of *F8* large deletion location with ITI outcome is unclear.
• We evaluated ITI outcomes in 34 children with severe hemophilia A carrying *F8* large deletions.
• Isolated A1-domain deletions were associated with better ITI outcomes.
• Exploratory analyses found residual *F8* transcripts and variable FVIII-related signals.

## 1 Introduction

Approximately 40% of pediatric patients with severe hemophilia A develop neutralizing antibodies (inhibitors) against factor VIII (FVIII), and among these, approximately 30% fail inhibitor eradication despite repeated high-dose FVIII infusion, a procedure known as immune tolerance induction (ITI). Failure to achieve inhibitor eradication is clinically defined as a manifestation of immune intolerance [1,2].

Deletion mutations, one of the common mutation types in pediatric severe hemophilia A, are regarded as the most deleterious for immune intolerance [[3], [4], [5]]. From a theoretical perspective, 5′/N-terminal *F8* deletions may be presumed to represent a high-risk subtype because 5′/N-terminal large deletions may disrupt essential regulatory or early coding regions, potentially resulting in absent endogenous FVIII expression and impaired immune tolerance [6,7]. In a hemophilia A mouse model, deletion of the *F8* promoter region and exon 1 resulted in undetectable F8 mRNA and the absence of FVIII expression, supporting the concept that disruption of the 5′/N-terminal *F8* regulatory/coding region may abolish endogenous FVIII production. However, it is important to note that this assumption is primarily derived from general principles of protein immunogenicity and mouse models rather than from direct clinical evidence in patients with hemophilia A [8]. To date, clinical data on the impact of different intragenic locations of *F8* deletions on ITI outcomes remain limited, particularly in children with severe hemophilia A.

However, conventional paradigms may not fully account for the intrinsic regulatory complexity of gene expression. Protein expression is typically controlled by genetic elements such as promoters and enhancers; therefore, a structurally altered *F8* gene may generate novel mRNA transcription patterns [9,10]. Activation of alternative promoters could potentially compensate for the loss of the canonical promoter, initiating residual FVIII expression that may influence immune tolerance, though this hypothesis has not yet been confirmed in pediatric severe hemophilia A [[11], [12], [13]].

Accordingly, we examined the association between the intragenic location of large *F8* deletions and ITI outcome in children with severe hemophilia A, with particular attention to deletions confined to the A1 domain. Exploratory molecular analyses were performed to assess residual *F8* expression in patients with available samples.

## 2 Methods

### 2.1 Study population and design

This single-center, retrospective cohort study was approved by the institutional review board of Beijing Children’s Hospital (BCH). Written informed consent for the collection and research use of clinical, genetic, and blood specimen data, including longitudinal follow-up and secondary analyses, was obtained from parents or legal guardians under an institutional hemophilia registry protocol (Approval No. [2025]-Y-255-D). Patients treated between January 2015 and December 2024 were included, and follow-up was updated through January 2026.

Inclusion criteria were as follows: (1) diagnosed with severe hemophilia A (FVIII: C <1%); (2) age below 18 years at the first clinical visit; (3) genetic testing confirmed the presence of a *F8* large deletion; (4) presence of FVIII inhibitors (at least 2 consecutive positive inhibitor titers, ≥0.6 Bethesda Units [BU]/mL); and (5) completion of ITI treatment with evaluable outcomes.

### 2.2 Data collection and laboratory methods

#### 2.2.1 Clinical data collection

Clinical data were retrospectively extracted from electronic medical records, including demographic information, baseline clinical characteristics, ITI treatment, and follow-up data.

#### 2.2.2 Genetic analysis

Genomic DNA was extracted from peripheral blood. Large deletions were detected using multiplex ligation-dependent probe amplification. Variants were classified according to the HGMD and local FVIII databases [14].

#### 2.2.3 Inhibitor monitoring and FVIII recovery testing

The inhibitor titer was measured at BCH using the Bethesda assay (Nijmegen modification) every 2 weeks during ITI until a steady inhibitor-titer decline was observed, and then monthly [15]. *In vivo* FVIII recovery was performed once the inhibitor titer was negative. After the *in vivo* FVIII recovery was ≥66%, monitoring frequency was reduced to every 3 months until the study end [16].

### 2.3 Definitions and patient stratification

#### 2.3.1 Definition of *F8* large deletions

*F8* large deletion was defined as an exon-level deletion involving one or more complete coding exons of *F8*, as identified by deletion/duplication analysis [17]. This operational definition included single-exon, multiexon, and whole-gene deletions but excluded intron 1 and intron 22 inversions and small intragenic sequence variants. Deleted exons were mapped to the corresponding FVIII coding domains according to the reference *F8* transcript (NM_000132.4) and protein sequence (NP_000123.1).

#### 2.3.2 Definition of *F8* domain location

FVIII domain boundaries were defined according to the canonical mature human FVIII protein sequence [18]. The protein comprises the A1 (residues 1–372), A2 (373–740), B (741–1648), A3 (1690–2019), C1 (2020–2172), and C2 (2173–2332) domains; the acidic a3 region spans residues 1649–1689 between the B and A3 domains. Deleted *F8* exon(s) were mapped to the corresponding FVIII coding domain(s). An isolated A1-domain deletion was defined as a deletion confined to exons encoding residues within the A1 domain, without extension into the A2, B, A3, C1, or C2 domains. All other deletion patterns were classified as nonisolated A1-domain deletions.

#### 2.3.3 ITI protocol

The ITI regimens and the selection of FVIII products (either recombinant or plasma-derived) were determined at the discretion of the managing clinician [19]. Patients primarily received either a low-dose regimen (FVIII 50 IU/kg 3×/wk) or an intermediate-dose regimen (FVIII 100 IU/kg/d).

#### 2.3.4 Outcome definition

• Success: achievement of both inhibitor elimination (FVIII inhibitor titer <0.6 BU/mL on 2 consecutive measurements) and normal FVIII recovery (≥66% of the expected value) within 24 months of treatment.
• Partial Success: inhibitor elimination with subnormal FVIII recovery (<66% of the expected value) within 24 months of treatment.
• Failure: failure to meet the criteria for Success or Partial Success within 24 months of treatment [19,20].

### 2.4 *F8* mRNA expression analysis by quantitative real-time polymerase chain reaction

Although liver sinusoidal endothelial cells are the principal source of circulating FVIII, obtaining liver tissue from pediatric participants solely for research purposes would be ethically unacceptable. Leukocyte-based analyses were therefore used as an accessible exploratory approach; similar approaches have been used in previous hemophilia studies to detect residual *F8* transcripts and intracellular FVIII-related signals [[21], [22], [23]]. However, these findings should not be interpreted as evidence of physiological FVIII production or a direct mechanism of immune tolerance.

Because patients with isolated A1-domain *F8* deletions showed relatively favorable ITI outcomes, molecular expression profiling was performed in this subgroup. We therefore performed molecular expression profiling in this subgroup to identify residual FVIII expression. Six such patients with eligible peripheral blood samples were enrolled, representing diverse ITI treatment responses. Peripheral blood samples were collected in sodium citrate anticoagulant tubes and centrifuged at 3000 rpm for 15 minutes. The cell-rich lower layer was isolated, and erythrocytes were removed by 2 rounds of lysis using 3 volumes of freshly prepared 1 × red blood cell lysis buffer. Total RNA was extracted from the resulting leukocyte pellets using TRIzol reagent (Invitrogen) and reverse-transcribed into cDNA using the PrimeScript RT Reagent Kit with gDNA Eraser (Takara). Domain-specific *F8* mRNA expression (A1, A2, A3, C1, and C2 domains; primers listed in Supplementary Table 1) was quantified by SYBR Green-based qPCR using TB Green Premix Ex Taq II (Takara, Cat. No. RR820A) on a LightCycler 480 II Real-Time PCR System (Roche). Expression levels were normalized to β-actin and calculated using the 2^–ΔΔCt method [24].

### 2.5 Protein expression analysis by Western blot

Total protein was extracted from leukocyte pellets using lysis buffer supplemented with 1% protease and phosphatase inhibitor cocktails. A total of 30 μg of protein lysate per sample was separated by 8% sodium dodecyl sulfate–polyacrylamide gel electrophoresis and transferred onto a polyvinylidene fluoride membrane. The membrane was incubated with a primary antibody against FVIII (monoclonal antibody, Cat. No. 66722-1-Ig, Proteintech) diluted at 1:5000, followed by a peroxidase-conjugated goat antimouse IgG secondary antibody (RS0001, Immunoway). The protein loading for each sample was normalized based on GAPDH expression. To prevent signal saturation due to disparate FVIII expression levels, protein loading in the healthy control group was titrated and reduced accordingly. Detection was carried out using an enhanced chemiluminescence system (Thermo Fisher Scientific).

### 2.6 Promoter activity analysis by dual-luciferase reporter assay

Potential promoter sequences within the A1 domain (chrX:154,194,417-154,250,827, hg19) were predicted using the online tool (https://www.fruitfly.org/seq_tools/promoter.html). From the 73 candidate sequences identified, the 10 highest-scoring candidate sequences were selected for functional validation using a dual-luciferase reporter assay (Supplementary Table 2).

To evaluate the transcriptional activity of the predicted promoter sequences, HEK293T cells were cultured in Dulbecco’s Modified Eagle Medium (DMEM) supplemented with 10% fetal bovine serum (FBS) and 1% penicillin-streptomycin (100×) at 37°C in a 5% CO_2_ humidified incubator. DMEM, FBS, penicillin-streptomycin, and 0.25% trypsin-EDTA were purchased from Gibco. For transfection, cells were plated in 24-well plates and transfected using PolyJet Transfection Reagent (SignaGen Laboratories, Cat. No. SL100688). Each well received 450 ng of the recombinant pGL4.15 firefly luciferase reporter plasmid containing a candidate promoter insert and 50 ng of the pRL-SV40 *Renilla* luciferase plasmid as an internal control. At 48 hours posttransfection, cells were harvested, and luciferase activities were quantified using the Dual-Luciferase Reporter Assay System (Promega, Cat. No. E1910) following the manufacturer’s protocol. Firefly luciferase signals were normalized to the corresponding *Renilla* luciferase values for each sample. All experiments were performed in triplicate and independently validated in 3 autonomous runs.

### 2.7 Statistical analysis

Statistical analyses were performed using IBM SPSS Statistics version 26.0. Categorical variables are presented as frequencies and percentages, and continuous variables as the median and interquartile range (IQR). The primary outcome was dichotomized as ITI success/partial success vs ITI failure. Categorical variables were compared using Fisher’s exact test, and continuous variables were compared using the Mann–Whitney U-test.

Variables with a univariate *P* value < .05 were entered into a multivariable binary logistic regression model to assess factors independently associated with ITI outcome. Results are reported as odds ratios (ORs) with 95% confidence intervals (CIs). A 2-sided *P* value < .05 was considered statistically significant.

For exploratory molecular assays, quantitative real-time polymerase chain reaction results were described at the individual-patient level. Western blot densitometry data are presented as mean ± standard deviation and were compared using pairwise 2-tailed Student’s *t*-tests. For dual-luciferase assays, the numbers above each bar indicate the *P* value vs the empty-vector control, calculated using 2-tailed Student’s *t*-tests.

We used the STROBE reporting guideline to draft this manuscript, and the STROBE reporting checklist is included in the supplementary materials.

## 3 Results

### 3.1 Study cohort and baseline clinical information

A total of 1680 children with hemophilia A were screened during the study period. Of these, 310 developed FVIII inhibitors. After exclusion of patients who did not receive ITI treatment, lacked an evaluable ITI outcome, or had unavailable genetic testing reports, 211 patients with evaluable ITI outcomes and confirmed genetic testing results were identified. Among these patients, 177 without large *F8* deletions were excluded. The final analytical cohort comprised 34 children with severe hemophilia A, inhibitors, large *F8* deletions, and evaluable ITI outcomes (Figure 1).

**Figure 1:**
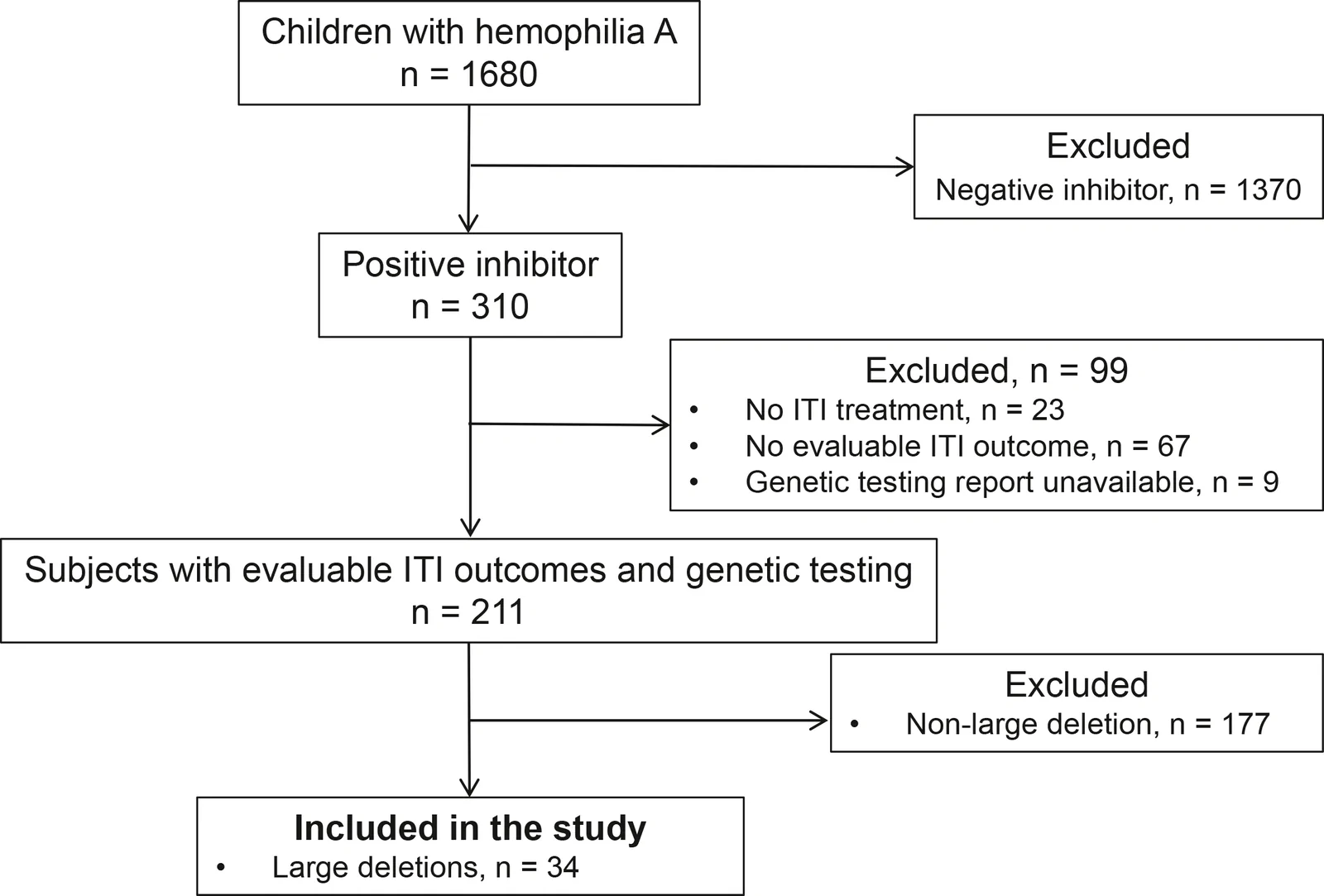
Flowchart of patient selection for the ITI outcome analysis. ITI, immune tolerance induction.

The 34 patients started ITI at the median age of 5.9 (IQR, 2.0-8.2) years, with historical peak inhibitor titer of 35.5 (IQR, 14.1-96.1) BU/mL, and a peak inhibitor titer during ITI of 81.0 (IQR, 29.3-264.6) BU/mL. Among enrolled patients, 9 patients (26.5%) achieved ITI success, 4 (11.8%) achieved partial success, and 21 (61.8%) experienced ITI failure, with follow-up duration after ITI-initiation of 28.3 months (IQR, 24.1–35.1 months) (Table 1).

**Table 1:** Baseline demographic and clinical characteristics of patients with *F8* large deletions undergoing ITI by outcomes.

Median (IQR) or N (%)	Large deletion cohort			
	Success/partial success	Failure	*P*^a^	*P*^b^
N, (%)	13 (38.2)	21 (61.8)		
Blood group				
A, Rh+	2 (15.4)	4 (19.0)	1.000	
B, Rh+	6 (46.2)	9 (42.9)		
AB, Rh+	1 (7.7)	1 (4.8)		
O, Rh+	4 (30.8)	7 (33.3)		
Family history of hemophilia				
Yes	3 (23.1)	4 (19.0)	1.000	
No	10 (76.9)	17 (81.0)		
Family history of inhibitors				
Yes	2 (15.4)	0 (0.0)	.139	
No	11 (84.6)	21 (100.0)		
Age at inhibitor diagnosis, (y)	2.1 (1.6-4.4)	4.5 (2.6-7.3)	.167	
Age at ITI initiation, (y)	2.1 (1.6-6.3)	7.0 (3.1-9.6)	.065	
Interval-time from inhibitor-diagnosis to ITI-initiation, (mo)	2.5 (1.0-7.5)	5.6 (0.7-41.8)	.436	
Exposure days before inhibitor-diagnosis, (d)	17 (13-23)	50 (14-55)	.141	
Maximum exposure days before inhibitor-diagnosis, (d)	3 (3-7)	7 (3-7)	.450	
Age at first hemorrhage, (m)	6.0 (5.0-10.0)	8.0 (6.0-12.0)	.444	
Age at first exposure, (m)	10 (6-14)	13 (7-25)	.227	
Type of first FVIII exposure				
Plasma-derived FVIII	12 (92.3)	16 (76.2)	.370	
Recombinant FVIII	1 (7.7)	5 (23.8)		
First exposure days, (d)	2 (1-5)	3 (2-6)	.264	
Joint hemorrhage before inhibitor development				
Yes	4 (30.8)	10 (47.6)	.477	
No	9 (69.2)	11 (52.4)		
ABR (from inhibitor-diagnosis to ITI-initiation)	9.6 (2.0-12.0)	9.6 (0.8-13.0)	.915	
AJBR (from inhibitor-diagnosis to ITI-initiation)	0.0 (0.0-8.0)	4.0 (0.0-8.0)	.404	
Preinhibitor treatment regimen				
On-demand	12 (92.3)	15 (71.4)	.210	
Prophylaxis	1 (7.7)	6 (28.6)		
Preinhibitor FVIII product type				
Plasma-derived FVIII	10 (76.9)	15 (71.4)	1.000	
Recombinant FVIII	3 (23.1)	6 (28.6)		
Historical peak inhibitor titer, BU/mL	8.4 (6.5-19.7)	69.4 (23.5-144.6)	.001	.081
Inhibitor titer pre-ITI, BU/mL	8.4 (2.7-89.0)	47.4 (22.0-168.8)	.012	.339
Peak inhibitor titer during ITI, BU/mL	28.5 (0.8-128.0)	142.9 (52.2-350.7)	.014	.614
ITI regimen				
Low-dose	10 (76.9)	17 (81.0)	1.000	
Intermediate-dose	3 (23.1)	4 (19.0)		
Type of FVIII for the ITI regimen				
Plasma-derived FVIII	6 (46.2)	17 (81.0)	.060	
Recombinant FVIII	7 (53.8)	4 (19.0)		
Mutation location				
Isolated A1-domain deletion	8 (61.5)	1 (4.8)	<.001	.035^c^
Nonisolated A1-domain deletion	5 (38.5)	20 (95.2)		

Low-dose ITI, FVIII 50 international units (IU)/kg 3×/wk; Intermediate-dose ITI, FVIII 100 IU/kg/d; Success: achievement of both inhibitor elimination (FVIII inhibitor titer <0.6 BU/mL on 2 consecutive measurements) and normal FVIII recovery (≥ 66% of the expected value) within 24 months of treatment; Partial Success: inhibitor elimination with subnormal FVIII recovery (<66% of the expected value) within 24 months of treatment; Failure: failure to meet the criteria for Success or Partial Success within 24 months of treatment. ABR, annual bleeding rate; AJBR, annual joint bleeding rate; BU, Bethesda units; CI, confidence interval; FVIII, factor VIII; IQR, interquartile range; ITI, immune tolerance induction; IU, international units; OR, odds ratio.

a *P* values were calculated using Fisher’s exact test for categorical variables and the Mann–Whitney U-test for continuous variables.

b Variables with univariate *P* values < .05 were entered into the multivariable binary logistic regression model.

c Adjusted OR, 21.59; 95% CI, 1.24–375.60; *P* = .035.

### 3.2 *F8* gene variant profile

Individual deletion profiles and corresponding ITI outcomes are summarized in Table 2. Among the 34 patients undergoing ITI, large *F8* deletions showed substantial heterogeneity in extent and distribution across the coding region (Table 2). Multiexon deletions predominated (29/34, 85.3%), whereas 5 patients (14.7%) carried single-exon deletions. Fourteen deletions (41.2%) were confined to a single FVIII domain, most commonly the A1 domain (9/34, 26.5%), while 20 deletions (58.8%) involved multiple domains. Based on the affected protein chains, 22 deletions (64.7%) were restricted to the heavy chain, 9 (26.5%) involved the light chain alone, and 3 (8.8%) spanned both chains. The number of amino acids encoded by the deleted exon(s) ranged from 41 to 2300 residues, with a median of 214 (IQR, 146-418) residues. Six patients (17.6%) had deleted exon(s) encoding more than 500 amino acids.

**Table 2:** Detailed genetic profiles of *F8* large deletions in patients undergoing immune tolerance induction.

Patient ID	ITI outcome	Deletion type	Deleted exon(s)	Affected FVIII domain(s)	Affected chain(s)	Amino acids encoded by deleted exon(s), n	>500 aa
P01	Success	Single-exon deletion	2	A1	Heavy	41	No
P02	Partial success	Multiexon deletion	2-3	A1	Heavy	81	No
P03	Partial success	Multiexon deletion	1-4	A1	Heavy	200	No
P04	Partial success	Multiexon deletion	1-4	A1	Heavy	200	No
P05	Success	Multiexon deletion	2-4	A1	Heavy	151	No
P06	Success	Multiexon deletion	2-6	A1	Heavy	214	No
P07	Failure	Multiexon deletion	2-6	A1	Heavy	214	No
P08	Success	Multiexon deletion	3-6	A1	Heavy	173	No
P09	Success	Single-exon deletion	7	A1	Heavy	73	No
P10	Failure	Multiexon deletion	2-8	A1/A2	Heavy	375	No
P11	Failure	Multiexon deletion	2-9	A1/A2	Heavy	433	No
P12	Partial success	Multiexon deletion	7-9	A1/A2	Heavy	218	No
P13	Failure	Multiexon deletion	8-9	A1/A2	Heavy	144	No
P14	Failure	Multiexon deletion	2-10	A1/A2	Heavy	464	No
P15	Failure	Multiexon deletion	3-10	A1/A2	Heavy	423	No
P16	Success	Multiexon deletion	7-11	A1/A2	Heavy	321	No
P17	Success	Single-exon deletion	11	A2	Heavy	71	No
P18	Failure	Multiexon deletion	2-12	A1/A2	Heavy	586	Yes
P19	Failure	Multiexon deletion	8-12	A1/A2	Heavy	297	No
P20	Failure	Multiexon deletion	10-12	A2	Heavy	153	No
P21	Failure	Multiexon deletion	1-13	A1/A2	Heavy	704	Yes
P22	Failure	Multiexon deletion	1-14	A1/A2/B/A3	Heavy + light	1739	Yes
P23	Failure	Multiexon deletion	15-20	A3/C1	Light	322	No
P24	Failure	Multiexon deletion	15-21	A3/C1	Light	351	No
P25	Failure	Multiexon deletion	19-21	A3/C1	Light	91	No
P26	Failure	Multiexon deletion	2-22	A1/A2/B/A3/C1	Heavy + light	2095	Yes
P27	Failure	Multiexon deletion	15-22	A3/C1	Light	403	No
P28	Failure	Multiexon deletion	21-22	C1	Light	80	No
P29	Success	Single-exon deletion	24	C2	Light	49	No
P30	Failure	Multiexon deletion	1-25	A1/A2/B/A3/C1/C2	Heavy + light	2300	Yes
P31	Failure	Multiexon deletion	23-25	C1/C2	Light	157	No
P32	Failure	Multiexon deletion	23-25	C1/C2	Light	157	No
P33	Failure	Single-exon deletion	26	C2	Light	51	No
P34	Success	Multiexon deletion	12-14	A2/B	Heavy	1155	Yes

Success: achievement of both inhibitor elimination (FVIII inhibitor titer <0.6 BU/mL on 2 consecutive measurements) and normal factor VIII (FVIII) recovery (≥ 66% of the expected value) within 24 months of treatment; Partial Success: inhibitor elimination with subnormal FVIII recovery (<66% of the expected value) within 24 months of treatment; Failure: failure to meet the criteria for Success or Partial Success within 24 months of treatment.

FVIII domain boundaries were defined according to the canonical mature human FVIII protein sequence. The protein comprises the A1 (residues 1–372), A2 (373–740), B (741–1648), A3 (1690–2019), C1 (2020–2172), and C2 (2173–2332) domains; the acidic a3 region spans residues 1649–1689 between the B and A3 domains. Deleted *F8* exon(s) were mapped to the corresponding FVIII coding domain(s). An isolated A1-domain deletion was defined as a deletion confined to exons encoding residues within the A1 domain, without extension into the A2, B, A3, C1, or C2 domains. All other deletion patterns were classified as nonisolated A1-domain deletions. A1, A1 domain; A2, A2 domain; A3, A3 domain; B, B domain; BU, Bethesda units; C1, C1 domain; C2, C2 domain; *F8*, coagulation factor VIII gene; FVIII, factor VIII; ITI, immune tolerance induction.

### 3.3 Association between *F8* deletion location and ITI outcome

Figure 2 depicts the exon-level landscape of large *F8* deletions and shows that ITI responses clustered predominantly among deletions confined to the A1 domain, whereas failures predominated among all other deletion patterns. Given this distribution and the structural boundaries of FVIII, mutations were categorized as isolated A1-domain vs nonisolated A1-domain deletions for subsequent univariate and multivariable analyses. Key demographic, clinical, treatment-related, laboratory, and mutation-related variables were then compared between patients with ITI success/partial success and those with ITI failure (Table 1).

**Figure 2:**
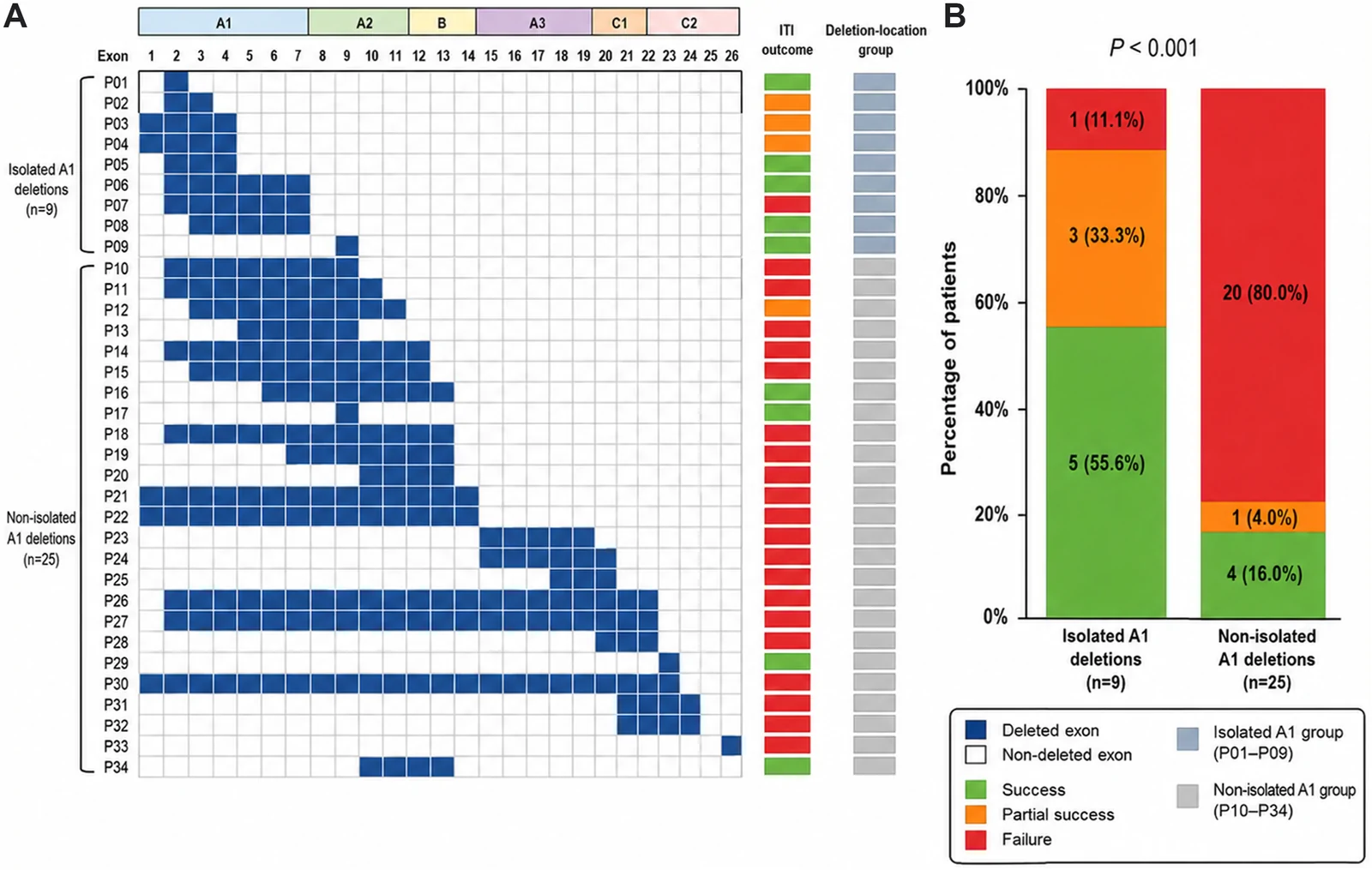
Exon-level landscape of large *F8* deletions and their relationship with ITI outcomes. Each row represents one patient, and each column represents an *F8* exon. Blue boxes indicate deleted exons, whereas white boxes indicate nondeleted exons. The upper annotation bar shows the corresponding factor VIII domains: A1, A2, B, A3, C1, and C2. Patients were categorized as having isolated A1-domain deletions or nonisolated A1-domain deletions. ITI outcomes are shown as success, partial success, or failure. Panel B compares the distribution of ITI outcomes between the 2 deletion-location groups. *P* values were calculated using Fisher’s exact test. ITI, immune tolerance induction; Success: achievement of both inhibitor elimination (factor VIII [FVIII] inhibitor titer <0.6 BU/mL on 2 consecutive measurements) and normal FVIII recovery (≥ 66% of the expected value) within 24 months of treatment; Partial Success: inhibitor elimination with subnormal FVIII recovery (<66% of the expected value) within 24 months of treatment; Failure: failure to meet the criteria for Success or Partial Success within 24 months of treatment.

Compared with the failure group, patients with success/partial success had lower historical peak inhibitor titers (8.4 [IQR, 6.5-19.7] vs 69.4 [IQR, 23.5-144.6] BU/mL; *P* = .001), pre-ITI titers (8.4 [IQR, 2.7-89.0] vs 47.4 [IQR, 22.0-168.8] BU/mL; *P* = .012), and peak titers during ITI (28.5 [IQR, 0.8-128.0] vs 142.9 [IQR, 52.2-350.7] BU/mL; *P* = .014). Isolated A1-domain deletions were observed more frequently among the success/partial success group (8/13 [61.5%] vs 1/21 [4.8%]; *P* < .001). Other assessed variables did not differ between groups.

Variables with *P* < .05 in the univariate analysis were entered into a multivariable binary logistic regression model. Deletion location was an independent predictor of ITI outcome. Compared with patients carrying non-A1 deletions, those with isolated A1-domain deletions had significantly higher odds of achieving ITI success or partial success (adjusted OR, 21.59; 95% CI, 1.24-375.60; *P* = .035).

### 3.4 FⅧ expression in patients with large deletions

To exploratorily assess residual *F8* expression in patients with isolated A1-domain deletions, we analyzed *F8* mRNA expression and FVIII-related immunoreactive signals in 6 individuals with available samples. Using primers specific to the A1, A2, A3, C1, and C2 domains, we detected variable residual mRNA expression. The expression profiles exhibited marked heterogeneity among patients with isolated A1-domain deletions (Figure 3). The single patient with ITI failure (P07) showed lower overall transcript levels than patients with ITI success or partial success.

**Figure 3:**
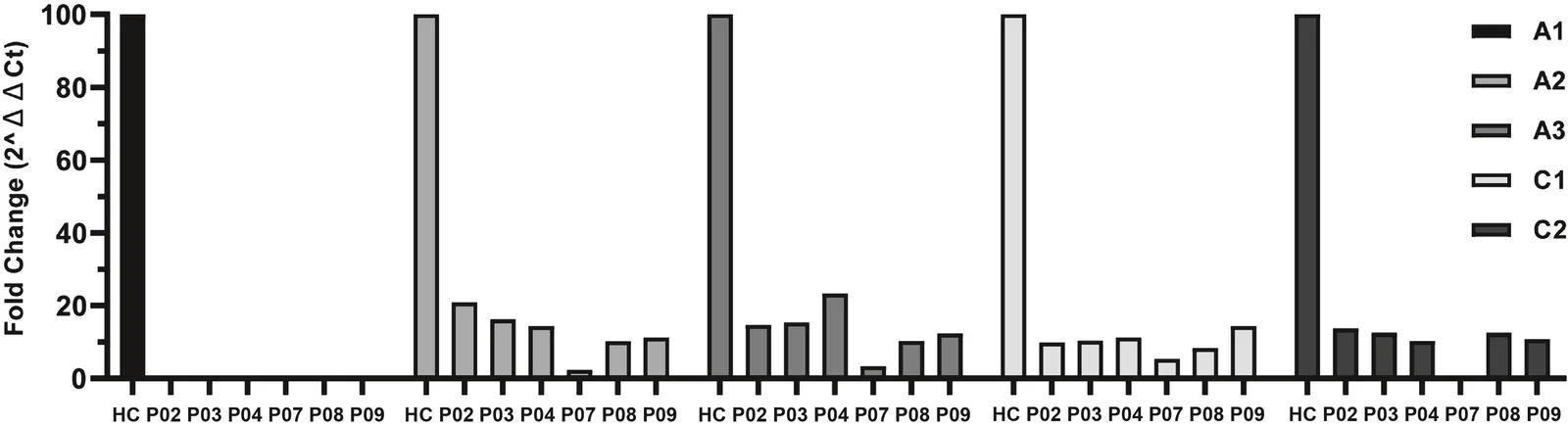
Relative *F8* mRNA expression in 6 patients with isolated A1-domain deletions. Total RNA was extracted from peripheral blood leukocyte pellets after erythrocyte lysis. *F8* mRNA levels were quantified by quantitative real-time PCR using domain-specific primers (A1, A2, A3, C1, C2; primers listed in Supplementary Table 1) and normalized to β-actin. Data are presented as relative expression (2^-^ΔΔCt method). The 6 patients (P02, P03, P04, P07, P08, P09) all carried isolated A1-domain deletions. Their ITI outcomes were as follows: P08, P09—success; P02, P03, P04—partial success; P07—failure. Among these, the patient who failed ITI (P07) showed substantially lower overall mRNA expression compared with those who achieved success or partial success. Ct, cycle threshold; *F8*, coagulation factor VIII gene; HC, healthy control; ITI, immune tolerance induction; mRNA, messenger RNA; PCR, polymerase chain reaction.

In exploratory Western blot analyses, FVIII-related immunoreactive signals were more prominent in patients with isolated A1-domain deletions who achieved ITI success than in the other groups (Figure 4). Given the small sample size and the use of leukocyte-derived lysates, these findings should be interpreted as exploratory.

**Figure 4:**
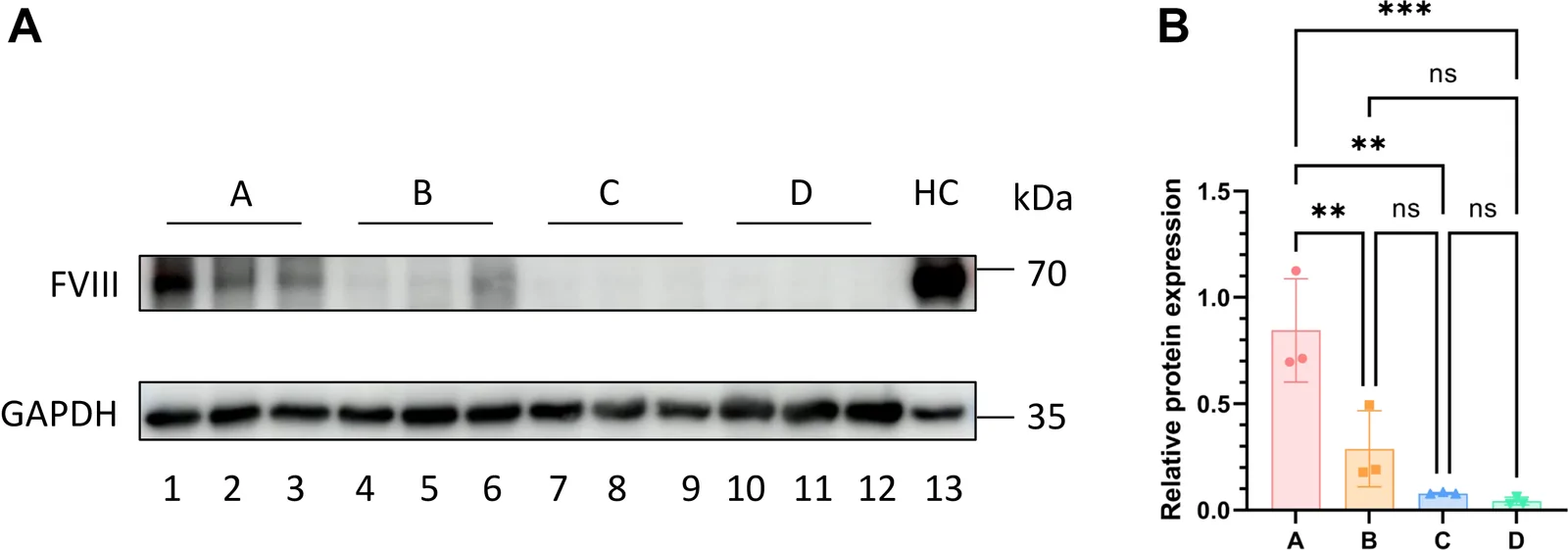
Expression analysis of the factor VIII (FVIII) protein in patient-derived samples with isolated A1-domain deletions or nonisolated A1-domain deletions. (A) Representative Western blot of the FⅧ protein and the GAPDH loading control. Whole-cell lysates, prepared from erythrocyte-lysed blood cells of patients and a healthy control, were separated on an 8% SDS-PAGE gel and immunoblotted with antibodies against the FⅧ protein and GAPDH. The lanes are as follows: Lane numbers (1-13) are indicated above each lane in the figure. Lanes 1-3, patients with isolated A1 deletion and a successful outcome, defined as group A. Lanes 4-6, patients with isolated A1 deletion and partial success outcome, defined as group B. Lanes 7-9, patients with nonisolated A1 deletion and success outcomes, defined as group C. Lanes 10-12, patients with nonisolated A1 deletion and failure outcomes, defined as group D. Lane 13, HC. The expected molecular weights for the FⅧ protein are indicated. GAPDH served as an internal loading control. (B) Densitometric quantification of FVIII-related immunoreactive signals normalized to GAPDH. Data are presented as the mean ± standard deviation of 3 patient samples per group (n = 3). Statistical methods are described in the Methods section. ∗∗, *P* < .01; ∗∗∗, *P* < .001; ns, not significant. FVIII, factor VIII; GAPDH, glyceraldehyde-3-phosphate dehydrogenase; HC, healthy control; ns, not significant; SDS-PAGE, sodium dodecyl sulfate–polyacrylamide gel electrophoresis.

### 3.5 Verification of predicted promoter activities

Bioinformatic screening was performed to identify potential promoter sequences within the A1-domain region that might support alternative transcriptional initiation. A total of 73 sequences with potential promoter activity within the A1 domain were predicted using bioinformatic tools (Supplementary Table 2). The 10 highest-scoring sequences were evaluated using dual-luciferase reporter assays. Among the tested sequences, only sequence 7 showed a statistically significant increase in luciferase activity compared with the empty-vector control (1.48-fold; 95% CI, 1.34-1.62; *P* = .0046). Sequence 9 showed a 1.15-fold increase but was not statistically significant (95% CI, 0.47-1.83; *P* = .4414). The remaining sequences showed no increase in luciferase activity relative to the empty-vector control; where statistically significant differences were observed, activity was reduced rather than increased (Figure 5). The modest increase observed for sequence 7 should be interpreted as an exploratory reporter-assay signal and does not establish physiologically relevant promoter activity, endogenous *F8* transcription, or a causal mechanism for immune tolerance.

**Figure 5:**
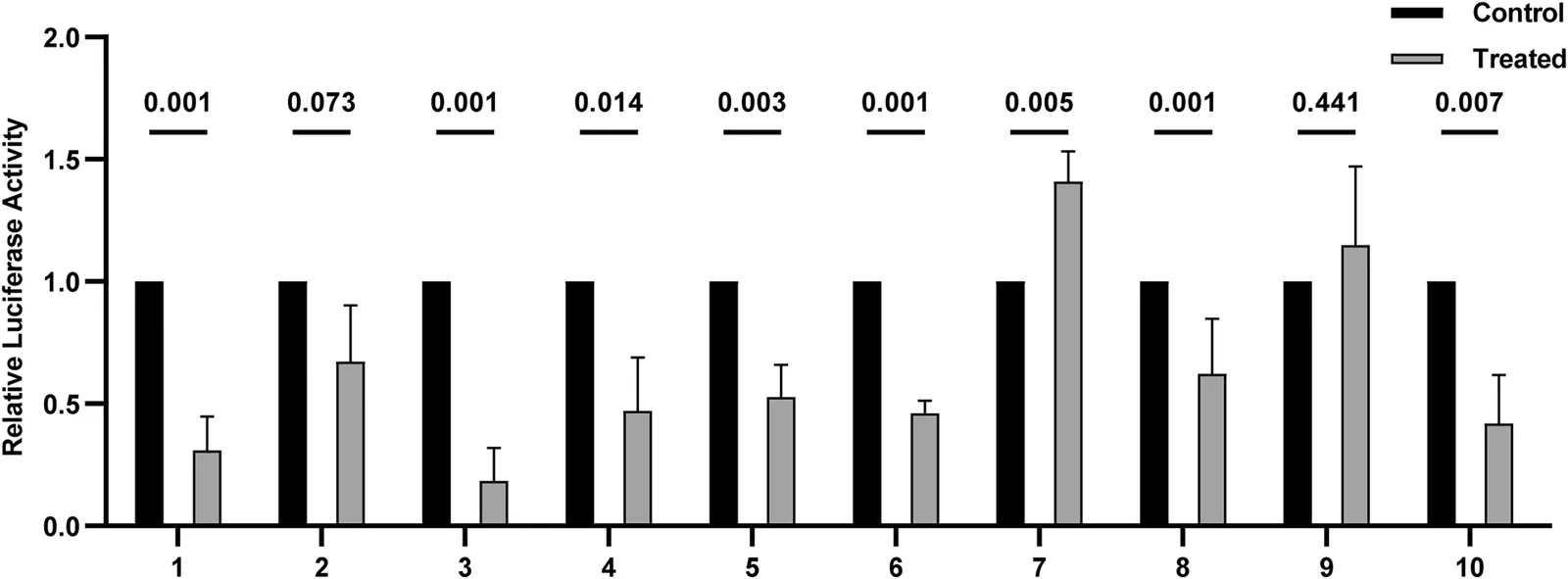
Dual-luciferase assay of predicted promoter sequences within the A1 domain of the *F8* gene. Ten candidate sequences (numbered 1–10) with the highest bioinformatic prediction scores for promoter activity were tested (see Supplementary Table 2 for sequence details and genomic coordinates). Each candidate sequence was cloned upstream of the firefly luciferase reporter gene in the pGL4.15 vector. HEK293T cells were cotransfected with the reporter construct and the pRL-SV40 Renilla luciferase control plasmid. Firefly luciferase activity was normalized to Renilla luciferase activity and is presented as mean fold-change relative to the empty-vector control (pGL4.15 without insert) ± standard deviation from 3 independent experiments performed in triplicate. Numbers above the bars indicate *P* values for comparison with the empty-vector control, calculated using two-tailed Student’s *t*-tests. Sequence 7 showed a modest increase in luciferase activity relative to the empty-vector control (1.48-fold; 95% CI, 1.34-1.62; *P* = .0046). Sequence 9 showed a 1.15-fold increase but was not statistically significant (95% CI, 0.47-1.83; *P* = .4414). The remaining sequences showed no increase in luciferase activity relative to the empty-vector control; where statistically significant differences were observed, activity was reduced rather than increased. CI, confidence interval; *F8*, coagulation factor VIII gene; HEK293T, human embryonic kidney 293T; pGL4.15, pGL4.15 luciferase reporter vector; pRL-SV40, pRL-SV40 Renilla luciferase control plasmid.

## 4 Discussion

In HA, *F8* gene mutations are important determinants of immune tolerance outcomes, with deletions in the *F8* gene being associated with an unfavorable ITI prognosis in patients with severe hemophilia A [25,26]. This study observed that ITI outcomes varied according to the intragenic location of *F8* large deletions. In particular, patients with isolated A1-domain deletions had more favorable ITI outcomes than those with nonisolated A1-domain deletions.

Traditionally, large deletions have been uniformly classified as a high-risk mutation category [25]. Such mutations, which may result in absent or markedly reduced endogenous FVIII expression, are considered challenging with respect to ITI efficacy [27,28]. However, whether clinical outcomes differ according to the intragenic location of *F8* large deletions remains unclear, as systematic studies are lacking and direct evidence is limited. Accordingly, we explored the association between the location of *F8* large deletions and ITI outcome in children with severe hemophilia A, rather than comparing different *F8* mutation classes. Our previous work has systematically characterized the *F8* mutation spectrum in Chinese children with severe hemophilia A and suggested an association between mutation type and ITI outcomes [4,14]. We have also previously proposed that trace FVIII expression might confer an immunoprotective effect [29]; however, the systematic link between location of mutation, trace expression, and clinical phenotype has not yet been explored for specific mutation types such as large deletions. The present study evaluated the association between deletion location and ITI outcome in a clinical cohort. To explore potential biological correlates, we integrated retrospective clinical data with prospective exploratory experiments, aiming to relate clinical phenotypes to patterns of *F8* expression.

In this retrospective cohort of 34 children with severe hemophilia A carrying *F8* large deletions, 9 (26.5%) patients achieved ITI success. This finding is broadly consistent with previous studies exploring the association between *F8* genotype and ITI outcome. Reported ITI success rates among patients with *F8* large deletions have ranged from 16.7% to 30.8%. Differences in outcome definitions and patient characteristics across studies may account for this variation [5,26,30]. Nevertheless, all available studies have suggested that *F8* large deletions are associated with less favorable ITI outcomes. The exon-level deletion map showed substantial heterogeneity in deletion extent and location. Notably, patients with isolated A1-domain deletions were more likely to achieve ITI success or partial success than those with nonisolated A1-domain deletions (8/9 vs 5/25). This association remained significant after adjustment for relevant clinical and inhibitor-related variables, suggesting that the intragenic location of *F8* large deletions may provide additional prognostic information for ITI outcome beyond the conventional classification of large deletions as a uniformly high-risk mutation category.

Among patients with isolated A1-domain deletions, residual *F8* transcript levels appeared to vary according to ITI outcome; the lowest overall level was observed in the single patient with ITI failure. In the exploratory protein analysis, FVIII-related signals were highest in 3 patients with isolated A1-domain deletions who achieved ITI success, and minimal or undetectable in patients with nonisolated A1-domain deletions, irrespective of ITI outcome. Although leukocytes are not the primary site of FVIII synthesis, they have been used previously to detect residual *F8* transcripts in HA patients [22,23,28]. These findings should therefore be interpreted as exploratory and do not establish systemic FVIII production or a causal mechanism for ITI outcome. Nevertheless, the observed expression patterns are consistent with the possibility that residual endogenous *F8* expression may contribute to interindividual variation in immune tolerance. Residual endogenous FVIII expression could theoretically provide limited self-antigen exposure during immune development and thereby contribute to immune tolerance [31]; however, this possibility remains speculative in the present study.

Previous studies have suggested that some variants classified as nonfunctional may retain trace FVIII expression and that intracellularly synthesized, unsecreted FVIII could potentially contribute to immune tolerance [13,32]. In patients with intron 22 inversion, truncated FVIII products derived from *F8B* and antisense transcripts have also been proposed to support central tolerance [10,33]. However, this hypothesis remains controversial, as a recent study failed to detect FVIII in humans and dogs with intron 22 inversion [34]. Therefore, the relevance of residual *F8* transcription or FVIII-related signals to inhibitor biology and immune tolerance remains uncertain.

In the present study, residual *F8* transcripts were detected in patients with isolated A1-domain deletions. These findings provide exploratory support for the possibility of residual expression but do not establish physiological FVIII production or a causal relationship with ITI outcome. Further studies using physiologically relevant models are needed to clarify whether residual *F8* expression contributes to immune tolerance. In addition, we performed a preliminary dual-luciferase screen of bioinformatically predicted sequences within the A1 domain. Although the exploratory design limits definitive interpretation of promoter function, the modest reporter signal observed for sequence 7 was directionally consistent with the residual *F8* transcript and FVIII-related signal findings in patients with isolated A1-domain deletions. These observations provide a rationale for future studies using more comprehensive promoter validation strategies and physiologically relevant *F8* regulatory models.

This study has several limitations. First, this is a retrospective, single-center study with a small sample size. However, BCH receives patients from across China, providing some geographic diversity. The rarity of hemophilia A and the highly selected subgroup of children with severe hemophilia A and inhibitors, carrying *F8* large deletions who underwent evaluable ITI, inevitably limited the sample size. Fisher’s exact test was used for small-sample categorical comparisons. Although univariate and multivariable analyses were performed to account for measured confounding factors, unmeasured confounding cannot be fully excluded. In addition, because only patients with evaluable ITI outcomes were included, selection bias cannot be completely excluded. However, the distribution of *F8* deletion location did not differ significantly between excluded and included patients (data not shown). Second, strict timeliness requirements for fresh blood samples confined molecular expression analysis to a subset of pediatric patients harboring *F8* large deletions. Such small-sample results on residual FVIII expression are only exploratory rather than conclusive. Although residual mRNA and protein were detected, whether these transcripts are effectively translated into immunologically relevant peptides requires confirmation using more sensitive techniques such as mass spectrometry. Third, detection of *F8* mRNA and FVIII-related immunoreactive signals in blood-cell pellets (primarily leukocytes) may not fully reflect physiologically relevant FVIII antigen presentation in liver sinusoidal endothelial cells, the principal source of circulating FVIII. Although leukocytes have been used in prior hemophilia studies to detect residual transcripts, the relevance to immune tolerance mechanisms remains uncertain [[21], [22], [23]]. Future studies addressing these questions could deepen understanding of immune regulation in deletion mutations and inform the development of novel tolerance induction strategies.

In conclusion, in this retrospective cohort of children with severe hemophilia A carrying *F8* large deletions, deletion location was associated with ITI outcome. Patients with isolated A1-domain deletions had more favorable ITI outcomes than those with nonisolated A1-domain deletions. Exploratory molecular findings suggested residual *F8* expression in a subset of patients with isolated A1-domain deletions; however, its biological relevance and relationship to immune tolerance require further investigation. These findings support consideration of the intragenic deletion location when evaluating ITI prognosis in this specific patient population.

## References

[1] Hoots W.K. (2008). Arthropathy in inhibitor patients: differences in the joint status. Semin Hematol.

[2] Walsh C.E., Soucie J.M., Miller C.H. (2015). Impact of inhibitors on hemophilia A mortality in the United States. Am J Hematol.

[3] Di Minno G., Coppola A., Margaglione M., Rocino A., Mancuso M.E., Tagliaferri A. (2022). Predictors of inhibitor eradication by primary immune tolerance induction in severe haemophilia A with high responding inhibitors. Haemophilia.

[4] Sun J., Li Z., Huang K., Ai D., Li G., Xie X. (2022). F8 gene mutation spectrum in severe hemophilia A with inhibitors: a large cohort data analysis from a single center in China. Res Pract Thromb Haemost.

[5] Zuccherato L.W., Souza R.P., Camelo R.M., Dias M.M., Jardim L.L., Santana M.A.P. (2024). Large deletions and small insertions and deletions in the factor VIII gene predict unfavorable immune tolerance induction outcome in people with severe hemophilia A and high-responding inhibitors. Thromb Res.

[6] D’haene B., Attanasio C., Beysen D., Dostie J., Lemire E., Bouchard P. (2009). Disease-causing 7.4 kb cis-regulatory deletion disrupting conserved non-coding sequences and their interaction with the FOXL2 promotor: implications for mutation screening. PLoS Genet.

[7] Kleinjan D.A., van Heyningen V. (2005). Long-range control of gene expression: emerging mechanisms and disruption in disease. Am J Hum Genet.

[8] Luo S., Li Z., Dai X., Zhang R., Liang Z., Li W. (2021). CRISPR/Cas9-mediated in vivo genetic correction in a mouse model of hemophilia A. Front Cell Dev Biol.

[9] Levinson B., Kenwrick S., Gamel P., Fisher K., Gitschier J. (1992). Evidence for a third transcript from the human factor VIII gene. Genomics.

[10] Pandey G.S., Yanover C., Miller-Jenkins L.M., Garfield S., Cole S.A., Curran J.E. (2013). Endogenous factor VIII synthesis from the intron 22-inverted F8 locus may modulate the immunogenicity of replacement therapy for hemophilia A. Nat Med.

[11] Babai A., Oliveira D., Gialeli A., Drozniewska M., Kainov Y., Dias C. (2025). Disruption of SPECC1L translation initiation by intragenic deletion: novel pathogenic mechanism in Teebi-hypertelorism syndrome. NPJ Genom Med.

[12] Famà R., Borroni E., Merlin S., Airoldi C., Pignani S., Cucci A. (2021). Deciphering the Ets-1/2-mediated transcriptional regulation of F8 gene identifies a minimal F8 promoter for hemophilia A gene therapy. Haematologica.

[13] Sauna Z.E., Lozier J.N., Kasper C.K., Yanover C., Nichols T., Howard T.E. (2015). The intron-22-inverted F8 locus permits factor VIII synthesis: explanation for low inhibitor risk and a role for pharmacogenomics. Blood.

[14] Sun J., Li Z., Li G., Huang K., Ai D., Liu G. (2022). Low-dose immune tolerance induction in children with severe hemophilia A with high-titer inhibitors: type of factor 8 mutation and outcomes. Res Pract Thromb Haemost.

[15] Verbruggen B., Novakova I., Wessels H., Boezeman J., van den Berg M., Mauser-Bunschoten E. (1995). The Nijmegen modification of the Bethesda assay for factor VIII:C inhibitors: improved specificity and reliability. Thromb Haemost.

[16] Björkman S. (2010). Limited blood sampling for pharmacokinetic dose tailoring of FVIII in the prophylactic treatment of haemophilia A. Haemophilia.

[17] Konkle B.A., Nakaya Fletcher S., Adam M.P., Bick S., Mirzaa G.M. (1993). GeneReviews®.

[18] Shen B.W., Spiegel P.C., Chang C.H., Huh J.W., Lee J.S., Kim J. (2008). The tertiary structure and domain organization of coagulation factor VIII. Blood.

[19] Li Z., Li Z., Cheng X., Zhang H., Yang C., Xu Q. (2025). Intermediate-dose immune tolerance induction outperforms with faster success, less bleeding, and no added cost in comparison with low dose: a multicenter randomized clinical trial. Res Pract Thromb Haemost.

[20] Ettingshausen C.E., Kreuz W. (2013). The immune tolerance induction (ITI) dose debate: does the International ITI Study provide a clearer picture?. Haemophilia.

[21] Castaman G., Giacomelli S.H., Mancuso M.E., Sanna S., Santagostino E., Rodeghiero F. (2010). F8 mRNA studies in haemophilia A patients with different splice site mutations. Haemophilia.

[22] Donadon I., McVey J.H., Garagiola I., Branchini A., Mortarino M., Peyvandi F. (2018). Clustered F8 missense mutations cause hemophilia A by combined alteration of splicing and protein biosynthesis and activity. Haematologica.

[23] Dutta D., Gunasekera D., Ragni M.V., Pratt K.P. (2016). Accurate, simple, and inexpensive assays to diagnose F8 gene inversion mutations in hemophilia A patients and carriers. Blood Adv.

[24] Schmittgen T.D., Livak K.J. (2008). Analyzing real-time PCR data by the comparative C(T) method. Nat Protoc.

[25] Garagiola I., Palla R., Peyvandi F. (2018). Risk factors for inhibitor development in severe hemophilia A. Thromb Res.

[26] Oomen I., Abdi A., Broer L., Camelo R.M., Callado F.M.R.A., Carvalho L.E.M. (2025). Large deletions in the F8 gene predict immune tolerance induction failure in people with severe hemophilia A. Res Pract Thromb Haemost.

[27] Atik T., Işık E., Onay H., Akgün B., Shamsali M., Kavaklı K. (2020). Factor 8 gene mutation spectrum of 270 patients with hemophilia A: identification of 36 novel mutations. Turk J Haematol.

[28] Gouw S.C., van den Berg H.M., Oldenburg J., Astermark J., de Groot P.G., Margaglione M. (2012). F8 gene mutation type and inhibitor development in patients with severe hemophilia A: systematic review and meta-analysis. Blood.

[29] Lu Y., Chen Z., Dai J., Wu X., Gu H., Li Z. (2020). Maternal microchimerism protects hemophilia A patients from inhibitor development. Blood Adv.

[30] Coppola A., Margaglione M., Santagostino E., Rocino A., Grandone E., Mannucci P.M. (2009). Factor VIII gene (F8) mutations as predictors of outcome in immune tolerance induction of hemophilia A patients with high-responding inhibitors. J Thromb Haemost.

[31] Starr T.K., Jameson S.C., Hogquist K.A. (2003). Positive and negative selection of T cells. Annu Rev Immunol.

[32] Spena S., Garagiola I., Cannavò A., Mortarino M., Mannucci P.M., Rosendaal F.R. (2018). Prediction of factor VIII inhibitor development in the SIPPET cohort by mutational analysis and factor VIII antigen measurement. J Thromb Haemost.

[33] Naylor J., Brinke A., Hassock S., Green P.M., Giannelli F. (1993). Characteristic mRNA abnormality found in half the patients with severe haemophilia A is due to large DNA inversions. Hum Mol Genet.

[34] Vir P., Gunasekera D., Dorjbal B., McDaniel D., Agrawal A., Merricks E.P. (2024). Lack of factor VIII detection in humans and dogs with an intron 22 inversion challenges hypothesis regarding inhibitor risk. J Thromb Haemost.

