# Supplementary material for: Isolated A1-domain F8 large deletions are associated with favorable immune tolerance induction outcomes in children with severe hemophilia A
Source: Res Pract Thromb Haemost. 2026 Aug 27;10(6):106927. doi: 10.1016/j.rpth.2026.106927 (PMC13641050; doi:10.1016/j.rpth.2026.106927)
Supplement: Supplementary file 1 [file mmc1.docx]

**Table S1. Primers used for the amplification of *F8***

| **Primer name** | **Forward primer** | **Reverse primer** |
| --- | --- | --- |
| A1 | ATCTCGGTGAGCTGCCTGTG | GCCTTGGCTTAGCGATGTTGAA |
| A2 | GTCCGATTTATGGCATACACAGATGAAAC | GGTAGATGTTATATGGTCTGCTTGCTTGA |
| A3 | AGGCTCTGGGATTATGGGATGAGTAG | CACGGTATAAGGGCTGAGTAAAGGAG |
| C1 | GATTACAGCTTCAGGACAATATGGACAGT | CTTGGTGCTCCAGGCATTGATTGA |
| C2 | TACTGCTCCTTCCATCTGCCTGAC | GCTTCCTCCAACTGCTCTATAACTTATCC |

**Table S2. Alternative promoter sequence**

| **No** | **start** | **end** | **score** | **promoter sequence** |
| --- | --- | --- | --- | --- |
| 1 | 2013 | 2063 | 1.00 | GAGGTGAACATATAAAAGGTGTTTAGGTCATAAGCGTTCCACCCTCATGA |
| 2 | 15386 | 15436 | 0.98 | GAAGAGATGCATATATAAGGAAGGGTATGTGGGAAGGGGCATGGGGCCAG |
| 3 | 16044 | 16094 | 1.00 | AAAAATATATATATATACTTTGGGAGGCCAAGGCGGGTGGATCACGAGGT |
| 4 | 39678 | 39728 | 0.96 | AAGGTGGGACTATATAGGTCCAGACAACTTGCCTCAATAGAGAACATCTC |
| 5 | 41111 | 41161 | 0.98 | ACAACACATATAAAAATGACTCTGGCAATTCACATCAGCTAGCCTCTGCA |
| 6 | 46702 | 46752 | 0.90 | AACTCATTTTATAAGAATAGTCAGCCGGGCGCGGTGGCTCACGCTTGTAA |
| 7 | 46838 | 46888 | 0.99 | TACTATCAGTATAAAAAATTAGCCAGGCGTGGTGGCGGGCGCCTGTCGTC |
| 8 | 47199 | 47249 | 0.98 | GGCTGATGGTTTTATAAAGGGCAGTTCTCCTGTACAATGCTCTCTTGTCT |
| 9 | 47346 | 47396 | 0.99 | CTCTTTCCTTTATAAATTACCCAGTCTCGGGTATGTCCTCATAGCAGCAT |
| 10 | 54239 | 54289 | 0.95 | AAGCAATGCATATAAAAAGCGAAACAGTAAACATCTTGAAGAAAATCTG |
